# Regulation of Mammalian Mitochondrial Dynamics: Opportunities and Challenges

**DOI:** 10.3389/fendo.2020.00374

**Published:** 2020-06-11

**Authors:** Rong Yu, Urban Lendahl, Monica Nistér, Jian Zhao

**Affiliations:** ^1^Department of Oncology-Pathology, Karolinska Institutet, Karolinska University Hospital Solna, Stockholm, Sweden; ^2^Department of Cell and Molecular Biology, Karolinska Institutet, Stockholm, Sweden

**Keywords:** mitochondrial dynamics, fission, fusion, apoptosis, mitophagy

## Abstract

Mitochondria are highly dynamic organelles and important for a variety of cellular functions. They constantly undergo fission and fusion events, referred to as mitochondrial dynamics, which affects the shape, size, and number of mitochondria in the cell, as well as mitochondrial subcellular transport, mitochondrial quality control (mitophagy), and programmed cell death (apoptosis). Dysfunctional mitochondrial dynamics is associated with various human diseases. Mitochondrial dynamics is mediated by a set of mitochondria-shaping proteins in both yeast and mammals. In this review, we describe recent insights into the potential molecular mechanisms underlying mitochondrial fusion and fission, particularly highlighting the coordinating roles of different mitochondria-shaping proteins in the processes, as well as the roles of the endoplasmic reticulum (ER), the actin cytoskeleton and membrane phospholipids in the regulation of mitochondrial dynamics. We particularly focus on emerging roles for the mammalian mitochondrial proteins Fis1, Mff, and MIEFs (MIEF1 and MIEF2) in regulating the recruitment of the cytosolic Drp1 to the surface of mitochondria and how these proteins, especially Fis1, mediate crosstalk between the mitochondrial fission and fusion machineries. In summary, this review provides novel insights into the molecular mechanisms of mammalian mitochondrial dynamics and the involvement of these mechanisms in apoptosis and autophagy.

## Introduction

Mitochondria are double membrane-bound organelles present in most eukaryotic cells. They are traditionally considered to function as the powerhouses of the cell, generating adenosine triphosphate (ATP) through oxidative phosphorylation, used for cellular chemical energy. Mitochondria have their own genome, known as mitochondrial DNA (mtDNA), which in mammalian cells contains 37 genes that are essential for normal mitochondrial function. The mitochondrial genome encodes 2 rRNAs, 22 tRNAs, and 13 protein subunits that are essential for the oxidative phosphorylation process. A reason why these particular genes are found in the mitochondrial genome is that the encoded proteins are highly hydrophobic and thus would have a problem entering the mitochondria (1–3). Besides these proteins, however, most (99%) of mitochondrial proteins in mammalian cells are encoded by nuclear genes, synthesized in the cytoplasm, and imported into mitochondria (4–7). In addition to producing ATP, mitochondria are involved in regulating numerous cellular activities, including cell-cycle progression and cell proliferation, the maintenance and differentiation of cells, mitophagy, and autophagy, the mitochondria-mediated intrinsic cell-death pathway and transduction of calcium signaling. Additionally, mitochondria are the major organelles generating and detoxifying reactive oxygen species (ROS) to adjust cellular redox homeostasis (8).

Mitochondria are highly dynamic and constantly alter their shape through fusion and fission events. The balance of fission and fusion events is termed *mitochondrial dynamics*, which is controlled by a number of mitochondria-shaping proteins encoded by genes in the nuclear genome. In addition to mitochondrial morphology, mitochondrial dynamics regulates the size, number, distribution, quality control, and transport of mitochondria in cells. Activated fission and/or inhibited fusion can lead to fragmented mitochondria, whereas active fusion and/or inhibited fission conversely can cause mitochondrial elongation (9–11). Over the last decades, emerging evidence indicates that deregulation of mitochondrial dynamics causes mitochondrial dysfunction, impacting on a broad range of cellular functions, and associated with a number of human diseases (11–17).

In this review, we discuss how mitochondrial fusion and fission is regulated by the mitochondria-shaping proteins, and by other co-factors such as the endoplasmic reticulum (ER) and the actin cytoskeleton, as well as how mitochondrial dynamics influences a variety of cellular biological processes such as apoptosis and mitophagy.

## The Mitochondrial Fusion Machinery

### The Yeast Mitochondrial Fusion Machinery

**Figure 1:**
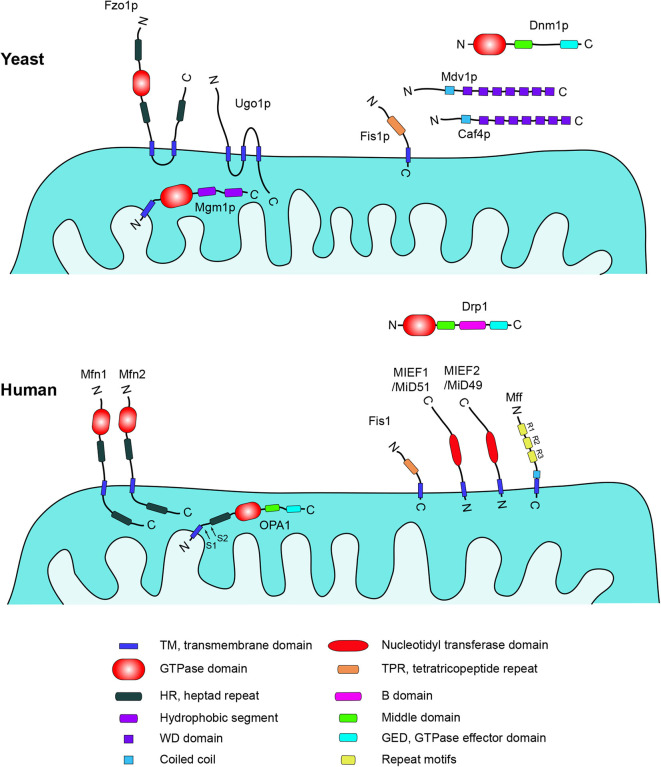
Schematic representation of structures, domains, and locations of key mitochondria-shaping proteins in yeast and human cells. Data were compiled from UniprotKB (http://www.uniprot.org). Yeast: Fzo1p (855aa, P38297), Ugo1p (502aa, Q03327), Mgm1p (881aa, P32266), Dnm1p (757aa, P54861), Mdv1p (714aa, P47025), Caf4p (643aa, P36130), Fis1p (155aa, P40515). Homo sapiens: Mfn1 (741aa, Q8IWA4), Mfn2 (757aa, O95140), OPA1 (960aa, O60313), Drp1 (736aa, O00429), Fis1 (152aa, Q9Y3D6), MIEF1/MiD51 (463aa, Q9NQG6), MIEF2/MiD49 (454aa, Q96C03), Mff (342aa, Q9GZY8). Literature references are provided in the main text.

Mitochondrial dynamics has been studied in yeast for many years. The mitochondrial fusion machinery characterized in yeast is composed of three key proteins, Fzo1p and Ugo1p, which are anchored in the mitochondrial outer membrane (MOM), and Mgm1p, localized in the mitochondrial inner membrane (MIM) (Figure 1). Depleting any of these proteins results in mitochondrial fragmentation (18–20).

Fzo1p, a large dynamin-related GTPase, is integrated in the MOM through two adjacent transmembrane (TM) domains near its C-terminus, and with a highly conserved N-terminal GTPase domain facing the cytoplasm. A functional Fzo1p is a prerequisite for fusion of the MOM (21, 22). Mgm1p is also a dynamin-related GTPase, anchored in the MIM via an N-terminal TM domain and a GTPase domain and two hydrophobic segments close to the C terminus, which are exposed in the intermembrane space. Mgm1p is required for both mitochondrial outer and inner membrane fusion, and disruption of the MGM1 gene can result in mitochondrial fragmentation and loss of mtDNA in a Dnm1p-dependent manner (23–25). Both Fzo1p and Mgm1p are evolutionally conserved from yeast to human. There are two homologs of Fzo1p in mammalian cells, termed mitofusin 1 (Mfn1) and mitofusin 2 (Mfn2), and the mammalian homolog of Mgm1p is known as OPA1 (26, 27).

Ugo1p was initially identified through the screening for yeast mutants that lost mtDNA in Dnm1p-dependent mitochondrial division (28). Ugo1p is a mitochondrial outer membrane-anchored protein and contains three TM domains in the middle region. The N-terminus faces the cytosol interacting with Fzo1p directly and the C-terminal domain is exposed to the intermembrane space for Mgm1p binding; thus Ugo1p acts as a molecular bridge between Fzo1p and Mgm1p in mitochondrial fusion (29, 30). Ugo1p is required for both outer and inner mitochondrial membrane fusion (31), and loss of Ugo1p causes mitochondrial fragmentation in yeast (28). Two recent studies identified the nuclear-encoded mitochondrial outer membrane protein SLC25A46 as the mammalian protein most similar to the yeast Ugo1p, but knockdown of SLC25A46 results in mitochondrial hyperfusion, indicating an opposite effect in human mitochondrial dynamics compared to the role of Ugo1p in yeast (32, 33). Yet other proteins are involved in the mitochondrial fusion process in yeast. For example, the F-box protein Mdm30p is essential for Fzo1p ubiquitylation and degradation after GTP hydrolysis in the late stage of outer membrane fusion to maintain fusion-competent mitochondria in yeast, and this step requires Ugo1p (34–36). Pcp1p and Ups1p are required for the processing of Mgm1p to control mitochondrial morphology (37, 38).

### The Mammalian Mitochondrial Fusion Machinery

**Figure 2:**
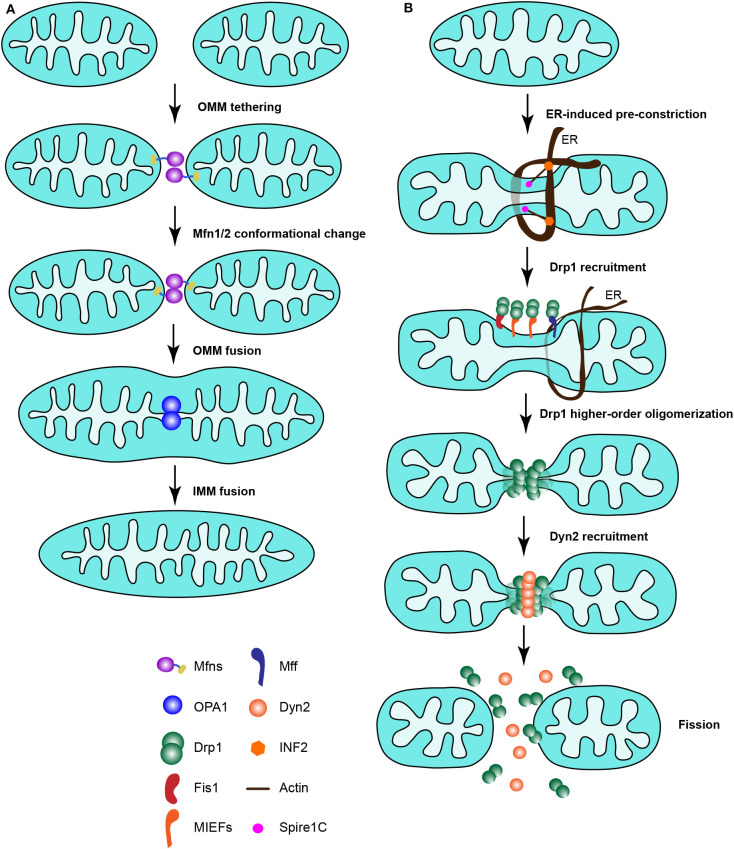
The mitochondrial fusion machinery and canonical Drp1-dependent mitochondrial fission machinery in mammals. **(A)** The mitochondrial fusion machinery. Two mitochondria are tethered and the MOM fused together by Mfn1/Mfn2, followed by fusion of the IMM by OPA1. **(B)** The canonical Drp1-dependent mitochondrial fission machinery in mammalian cells. Mitochondria are pre-constricted by the ER, together with actin filaments associated with mitochondria and the ER via Spire1C and INF2, respectively. At the ER constriction site, Drp1 is recruited by its receptors to mitochondria and assembled to form higher-order oligomers around the mitochondrial surface. Then mitochondria are further constricted by Drp1 oligomerization, and Dyn2 is instantly recruited to the constricted site to finalize the scission of mitochondria through GTP hydrolysis.

In mammals, there are three key dynamin-related proteins controlling mitochondrial fusion, mitofusin 1 (Mfn1), mitofusin 2 (Mfn2) and optic atrophy 1 (OPA1) (Figure 1). All of them are large dynamin-related GTPases and their activation proceeds via a three-step mitochondrial fusion process: Two mitochondria are tethered and form a docking ring structure around the contact point between the outer membranes. Then, the two outer membranes fuse together triggered by GTP hydrolysis, followed by a final fusion of the two inner membranes [(39, 40); Figure 2A].

Both mammalian Mfn1 and its paralog Mfn2 reside in the outer mitochondrial membrane. They are homologs of *Drosophila fzo*, and similar to the fusion protein Fzo1p in yeast. Based on the topology from the ortholog Fzo1p in yeast, it is generally accepted that both Mfns have a bipartite TM domain, with the N-terminal and C-terminal domains of the protein exposed toward the cytoplasm and with a GTPase domain close to the N-terminus (26). However, it has been demonstrated recently that Mfns in mammals only have a single TM domain, which makes the N-terminal GTPase and HR1 domains face the cytoplasm and the C-terminus with HR2 domain reside in the mitochondrial intermembrane space [(41); Figure 1]. Crystal structure studies prove that both Mfn1 and Mfn2 can form stable homodimers through their GTPase domains, which are critical for outer membrane fusion (42–44). Mfn1 and Mfn2 can also form heterodimers via GTPase domains in a nucleotide-dependent manner (42). Overexpression of either Mfn1 or Mfn2 can induce perinuclear mitochondrial clustering, whereas Mfn1/2-deficient cells contain severely fragmented mitochondria. Furthermore, Mfn1 and Mfn2 can form homo- and hetero-oligomers, and these three types of complexes can work in concert to tether the outer mitochondrial membranes of adjacent mitochondria (45). In spite of that both proteins are essential for the maintenance of mitochondrial morphology, Mfn1 has considerably higher GTPase activity than Mfn2 (46). In addition to controlling mitochondrial morphology, Mfn2 has other specific functions not observed for Mfn1. For instance, Mfn2 is involved in ER-mitochondria connections (47), energy metabolism and insulin signaling (48), mitophagy (49), and apoptosis (50). Strikingly, mutations in *MFN2* but not *MFN1* can cause Charcot-Marie-Tooth disease type 2A (CMT2A), a peripheral neuropathy characterized by axonal degeneration (51). However, Mfn1 (but not wild-type Mfn2) has been shown to compensate for mitochondrial fusion deficiency caused by *MFN2* mutations through the formation of Mfn1-Mfn2 heterooligomeric complexes (52) and rescues axonal degeneration induced by Mfn2 mutations (53). Along these lines, it is further shown that increased levels of Mfn1 expression in the nervous system prevents axonal degeneration in a CMT2A mouse model, suggesting a potential therapeutic strategy for this disease (54). Together, these data suggest that the two mammalian mitofusin proteins, Mfn1 and Mfn2, are functionally correlated but non-redundant.

OPA1 is the mammalian homolog of yeast Mgm1p. It is also a dynamin-related GTPase localized in the MIM and essential for fusion of the MIM. OPA1 was originally discovered by gene mutation screening of autosomal dominant optic atrophy (27). There are at least eight mRNA variants identified from the OPA1 gene through alternative splicing, generating long and short isoforms (55). Membrane–anchored long forms of OPA1 (L-OPA1) undergo proteolytic cleavage at the S1 or S2 sites by a group of proteases residing in the mitochondrial intermembrane space (e.g., PARL, PRELI, Yme1L, and OMA1), resulting in soluble short forms of OPA1 (S-OPA1) (13, 56–58). Recently, crystal structures of short Mgm1p (S-Mgm1) from *C. thermophilum* (59) and *S. cerevisiae* (60), orthologs of S-OPA1, have been determined, providing a mechanistic platform for understanding the functions of Mgm1/OPA1 during mitochondrial inner membrane fusion. It has been suggested that both long and short forms of OPA1 are required for inner membrane fusion (57). In contrast, there are reports suggesting that long forms of OPA1 are sufficient for mediating mitochondrial fusion when expressed in *Yme1l*^−/−^and *Oma1*^−/−^cells. This may indicate that Opa1 processing is not necessary for fusion, and that overexpression of short OPA1 forms trigger mitochondrial fragmentation in *Yme1l*^−/−^ cells (61). However, recent studies show that S-OPA1 play a role in MIM fusion, and cryo-electron microscopy (cryo-EM) studies reveal that S-OPA1 has a dynamin-like structure and employs dynamin-like power stroke membrane remodeling for MIM fusion (62). Moreover, moderate levels of S-OPA1 together with L-OPA1 are required for efficient and fast membrane pore opening during the fusion process, but excess levels of S-OPA1 inhibit fusion activity (63). OPA1 processing can also be affected by mitochondrial membrane potential and proapoptotic stimuli. For example, Caspase-3 can lead to N-terminal cleavage of OPA1 resulting in mitochondrial fragmentation (64), and CCCP treatment is also known to induce cleavage of long OPA1 forms by OMA1 (65, 66). Knockdown of OPA1 causes mitochondrial fragmentation (67), and overexpression of OPA1 induces mitochondrial elongation (68). Although Mfn1, Mfn2, and OPA1 are all essential for controlling mitochondrial fusion, overexpression of OPA1 can counteract the effect of Mfn2 knockout but not the effect of Mfn1 loss on mitochondrial morphology (68). This confirms that there is a functional difference between Mfn1 and Mfn2.

## The Mitochondrial Fission Machinery

### The Yeast Mitochondrial Fission Machinery

In yeast, four key proteins are involved in the mitochondrial division process: Dnm1p, Fis1p, Mdv1p, and Caf4p (Figure 1). The dynamin-related GTPase Dnm1p is a central component in the mitochondrial fission machinery, and was initially discovered by screening yeast mutants with defective mitochondrial morphology (69, 70). The re-localization of Dnm1p from the cytosol to mitochondria is a key step in mitochondrial fission. Fis1p is the mitochondrial receptor recruiting Dnm1p to the surface of mitochondria via one of the adaptors Mdv1p or Caf4p, which act as protein bridges between Fis1p and Dnm1p. Then Dnm1p self-assembles around the mitochondrial surface forming spiral-like structures at constriction sites leading to mitochondrial scission (71–74).

Fis1p is anchored in the MOM via the C-terminal tail and the N-terminal domain is facing the cytosol (75). The cytoplasmic domain of Fis1p contains a tetratricopeptide repeat (TPR) domain forming a concave surface, and a short N-terminal helix, which is required for binding and recruiting Mdv1p to the concave surface (76). Both Mdv1p and its paralog Caf4p are soluble cytosolic proteins containing an N-terminal extension, a middle coiled-coil domain, and a C-terminal WD repeat domain. Acting as molecular adaptors and bridges between Dnm1p and Fis1p, these proteins can bind to Dnm1p through the WD repeat domain and then associate with Fis1p through the N-terminal extension (74, 77).

In Dnm1p-null cells, when mitochondrial fission is blocked, mitochondria form long tubular networks and mitochondrial membranes collapse to one side of the cell (69). Fis1p-null mutation also causes mitochondrial reticular formation (75), indicating that both Dnm1p and Fis1p are critical for mitochondrial division in yeast. In yeast cells where only one of the Mdv1p or Caf4p genes has been deleted, mitochondrial morphology is similar to wild type, but in cells with both genes deleted, like in Fis1p-null mutant cells, mitochondria show an elongated and net-like morphology, and most of Dnm1p stays in the cytosol. Interestingly, overexpression of Mdv1p or Caf4p can also inhibit mitochondrial fission, possibly because overexpressed Mdv1p or Caf4p blocks the recruitment of Dnm1p to mitochondria (74, 78). Mdv1p and Caf4p are also endowed with unique functions. For instance, Mdv1p is more active than Caf4p in promoting fission, whereas Caf4p but not Mdv1p in association with Fis1p can determine the polarized localization of Dnm1p clusters on the mitochondrial surface after Dnm1p recruitment (79). Furthermore, a truncated form of Mdv1p lacking the N-terminal extension (that binds with Fis1p), fused with the TM domain of Tom20, can be tethered to the outer mitochondrial membrane, and this is sufficient to recruit Dnm1p to mitochondria and trigger fission in the absence of Fis1p (80).

### The Canonical Mitochondrial Fission Machinery in Mammals

#### Drp1—A Central Regulator of the Canonical Mitochondrial Fission Machinery

In mammals, the regulation of mitochondrial fission is considerably more complicated than in yeast. The dynamin-related protein 1 (Drp1) is the ortholog of yeast Dnm1p and shares 42% homology with Dnm1p (81). Structural analysis demonstrates that Drp1 exists in a dynamic equilibrium between multiple oligomeric states in cells, and the minimal functional assembly subunit is a dimer (82). Like its yeast ortholog, Drp1 acts as the master regulator and plays a central role in mammalian mitochondrial fission. In mammalian cells, Drp1 is primarily present in the cytosol, but can be recruited via any of its mitochondrial receptors (such as Fis1, Mff, MIEF1/MiD51, and MIEF2/MiD49, see Figure 1) to the mitochondrial surface, where it is assembled into higher-order complexes that wrap around the mitochondrial surface triggering mitochondrial fission through its GTPase activity (83). Thus, Drp1 and its four mitochondrial receptors Fis1, Mff, and MIEFs (MIEF1 and MIEF2) constitute the core components of the canonical mitochondrial fission machinery in mammals. In contrast to the strong evolutionary conservation between Drp1 and Dnm1p, their interacting factors, i.e., Mdv1p and Caf4p in yeast vs. Mff and MIEFs in mammals, are quite evolutionarily diverged: The yeast Mdv1p and Caf4p proteins have no mammalian homologs, whereas counterparts of mammalian Mff and MIEFs have not yet been identified in yeast (13). Even though mammalian Fis1 has been identified as the homolog of yeast Fis1p (84, 85), a growing body of evidence suggests that Fis1p and Fis1 have become functionally diverged in yeast and mammals (80, 86–88). Mammalian Fis1 is no longer an essential mitochondrial receptor responsible for recruitment of the cytosolic Drp1 to the mitochondrial surface. Instead, three additional mitochondrial proteins, Mff, MIEF1, and MIEF2, have been identified as major receptors for translocation of Drp1 to mitochondria in mammals (89–92). Another member of the dynamin-related family, Dynamin 2 (Dyn2) promotes membrane remodeling of multiple organelles (93). Recently, it was reported that Dyn2 is recruited to mitochondrial constriction sites transiently after Drp1 puncta accumulation and acts in the final step of Drp1-mediated mitochondrial division (94). However, a recent study suggests that although Dyn2 plays a role in mitochondrial fission, it is not essential for Drp1-mediated fission (95). Further supporting this notion, Drp1 is shown to be essential for mitochondrial and peroxisomal fission, whereas Dnm1, Dnm2 (Dyn2), and Dnm3 are dispensable (96).

Overall, during the mitochondrial fission process, Drp1 recruitment to mitochondria is a critical step, but the mechanisms underlying this process are not fully understood. A number of issues remain to be elucidated, for instance, why multiple mitochondrial receptors of Drp1 simultaneously exist in the cell; whether these receptors work independently of each other or in a coordinating way; and how the post-translational modifications and the oligomeric state of Drp1 impact on the process of mitochondrial division. Below, the different Drp1 receptors in mammals will be discussed in further detail.

#### The Mitochondrial Receptors for Drp1

In mammalian cells, the recruitment of Drp1 to mitochondria is mediated by four MOM-anchored proteins: fission protein 1 (Fis1), mitochondrial fission factor (Mff), mitochondrial elongation factor 1 (MIEF1/MiD51), and mitochondrial elongation factor 2 (MIEF2/MiD49).

##### Fis1

Mammalian Fis1 is the ortholog of yeast Fis1p and was initially identified as the receptor for the recruitment of Drp1 to mitochondria in mammals based on the studies of Fis1p. Like yeast Fis1p, mammalian Fis1 is localized to the MOM via its C-terminal TM domain, and with the N-terminal region of Fis1 facing the cytosol (84, 85). Structural analysis shows that the N-terminal region contains a TPR-like core domain, which is different from the typical TPR motif, but still important for binding to other proteins (97). In yeast, Fis1p acts as the receptor for the recruitment of Dnm1p (Drp1 in mammals) to mitochondria through the adaptors Mdv1p or Caf4p, thereby regulating mitochondrial morphology (98, 99). Since Fis1 is evolutionarily conserved from yeast to human, mammalian Fis1 was initially believed to have similar functions to its ortholog Fis1p in yeast, i.e., serving as the receptor for the recruitment of Drp1 to mitochondria, promoting fission. In keeping with a pro-fission role, several early studies showed that overexpression of human Fis1 (hFis1) results in extensive mitochondrial fragmentation, whereas knockdown of hFis1 causes mitochondrial elongation (84, 85, 100–102). However, due to the absence of yeast adaptors Mdv1p and Caf4p in mammals, whether hFis1 promotes mitochondrial fragmentation through a similar mechanism as in yeast has remained quite controversial. For example, it was reported that depletion of hFis1 induces mitochondrial elongation in HeLa cells and in Fis1-null mouse embryonic fibroblasts (MEFs) (86, 103, 104), but does not affect mitochondrial morphology in HCT116 cells (90). Furthermore, increased or decreased levels of Fis1 do not seem to affect the subcellular distribution of Drp1 between the cytosol and mitochondria in mammalian cells. For instance, elevated levels of hFis1 in 293T cells do not affect the subcellular distribution of Drp1, but promote mitochondrial fragmentation (91). Likewise, knockdown of hFis1 in HeLa and HCT116 cells does not reduce levels of Drp1 on mitochondria (90, 104). However, in Fis1-null MEFs, Drp1 puncta on mitochondria are reduced to some extent (86). Taken together, these studies suggest that mammalian Fis1 is not essential for Drp1 recruitment and Drp1-mediated mitochondrial fission, but might play specific roles in some physiological processes or certain types of mammalian cells.

In spite of the facts presented above, however, it is found that overexpression of Fis1 can induce extensive mitochondrial fragmentation in all mammalian cells analyzed. Given the absence of Mdv1p and Caf4p in mammals as well as a minor role of Fis1 in Drp1 recruitment and Drp1-dependent fission, the molecular mechansms underlying mammalian Fis1-induced mitochondrial fragmentation have remained enigmatic. Moreover, human Fis1 cannot rescue the mutant phenotype in yeast cells lacking Fis1p (100), while yeast Fis1p, when expressed in human HeLa cells, is targeted to mitochondria, but does not affect mitochondrial morphology (101). These data support the notion that despite similarities in protein structure, membrane topology and mitochondrial localization, Fis1p in yeast and hFis1 in humans have functionally diverged during evolution. Thus, the precise roles of hFis1 in regulating mitochondrial dynamics remain to be clarified.

However, despite the fact that hFis1 is dispensable for Drp1 recruitment to mitochondria in many conditions, it should be emphasized that hFis1 does play a role in regulating mitochondrial fission by recruiting Drp1 to mitochondria, especially in response to cell stress-induced mitochondrial fragmentation, such as mitophagy/apoptosis-related fission, or pathophysiology-associated fission (105–109). In normal conditions, the interaction between Fis1 and Drp1 is weak in mammalian cells, but the interaction can be enhanced after treatment with different apoptotic or autophagic stimuli, and is accompanied by mitochondrial fragmentation (91, 106, 110–112).

##### Mff

Mff was originally identified through a small interfering RNA (siRNA) screen in *Drosophila melanogaster* cells, and it exists in metazoans but not in yeast. The Mff gene generates at least nine different isoforms by alternative splicing (89). Similar to Fis1, Mff has a C-terminal TM domain, by which it is anchored in the MOM, while its N-terminal region facing the cytosol contains three short amino acid repeats (R1-R3 motifs) and a coiled-coil domain. The first 50 N-terminal residues containing R1 and R2 motifs are essential for Drp1 recruitment, and this is also the minimal region required for Drp1-Mff interaction (89, 90, 113). Depletion of Mff in HeLa cells (89, 90) or MEFs (86) severely inhibits mitochondrial fission and largely abrogates Drp1 recruitment to mitochondria, whereas overexpression of Mff in HeLa cells recruits most of Drp1 from the cytosol to mitochondria and induces extensive mitochondrial fission (90). It has become increasingly clear that Mff is the major receptor for Drp1 recruitment to mitochondria and thus actively promotes mitochondrial fission in mammals.

##### MIEF1 and MIEF2

MIEF1 and its paralog MIEF2 (also known as MiD51 and MiD49) were initially characterized by us and others as novel mitochondrial receptors for Drp1 recruitment to mitochondria in mammals (91, 92, 114). MIEF1 and MIEF2 are highly conserved vertebrate-specific mitochondrial proteins, not yet identified in invertebrates and plants (91, 114). Similar to the mitochondrial receptor Mff (89, 90), overexpression of either MIEF leads to extensive recruitment of the cytosolic Drp1 to mitochondria in a hFis1- and Mff-independent manner (91, 115). In contrast, MIEF overexpression induces mitochondrial elongation rather than fission in most of cells. Most likely, MIEFs act as Drp1 inhibitors sequestering it on the surface of mitochondria and inhibiting its GTPase activity (91, 92, 114, 115).

MIEF1 and MIEF2 are highly similar with respect to protein sequence, sharing 45% amino acid identity in human. Unlike hFis1 and Mff, both MIEF1 and MIEF2 have an N-terminal TM domain to anchor them in the MOM (91, 92, 114). Additionally, MIEF1 and MIEF2 can form homodimers and heterodimers (114). MIEF1 and MIEF2 however also differ in some aspects. For example, biochemical analysis shows that in addition to the monomeric form, MIEF1 appears predominantly as dimers, whereas MIEF2 appears as oligomers. Importantly, the first 1–49 residues including the TM domain are required for oligomerization of MIEF2, whereas the region between residues 109–154, but not the TM domain, is crucial for dimerization of MIEF1 (114). Moreover, their different crystal structures also indicate distinct functions. Both proteins have a nucleotidyl transferase domain, but MIEF1 can bind nucleotide diphosphates (ADP and GDP), while MIEF2 does not, and MIEF1 binding to ADP can stimulate Drp1 oligomerization, self-assembly and its GTPase activity (116–118). Interestingly, treatment of MIEF1- or MIEF2-overexpressing cells with antimycin A, an inhibitor of complex III of the electron transport chain, leads to mitochondrial fragmentation in cells overexpressing MIEF1 but not in cells overexpressing MIEF2. Moreover, MIEF1-induced fission requires ADP binding to MIEF1 in this process (86, 117). A recent cryo-EM structural analysis has revealed four interfaces of Drp1 that mediate the interaction with MIEFs, providing a structural basis for Drp1 recruitment to mitochondria (119).

## Interplay Between Different Drp1 Receptors in Mitochondrial Dynamics

An increasing number of studies have suggested that Mff and MIEFs play a significant role in recruitment of the cytosolic Drp1 to mitochondria in mammals, but the action of MIEFs seems to be more complicated than that of Mff in regulating mitochondrial fission and fusion. In general, it is observed that Mff overexpression leads to the extensive recruitment of Drp1 on mitochondria promoting mitochondrial fission, whereas depletion of Mff reduces Drp1 on mitochondria resulting in mitochondrial elongation. In contrast, the mechanisms by which MIEFs can regulate mitochondrial morphology remain puzzling with discordant results. For example, in 293T and HeLa cells, expression of exogenous MIEF1 or MIEF2 induces mitochondrial elongation. Both MIEF1 and MIEF2 can interact with and recruit Drp1 to mitochondria and distribute in a punctate manner on the mitochondrial surface (91, 114). In Cos-7 monkey cells, expression of exogenous MIEF1 or MIEF2 also leads to mitochondrial elongation and accumulation of Drp1 on mitochondria, however, at low levels of the MIEF protein, mitochondrial morphology is relatively normal (92). Further studies show that long-term expression of MIEF1 results in a variety of morphological changes of mitochondria from fragmentation to a network state, suggesting that low levels of exogenous MiD51/MIEF1 can increase fission events, whereas at high expression levels, fission events are inhibited, resulting in mitochondrial fusion (120). On the other hand, knockdown of MIEF1 and MIEF2 by siRNA leads to inconsistent effects on mitochondrial morphology. It was reported that in Cos-7 cells, knockdown of both MIEF genes is required to cause a mitochondrial fusion phenotype (92). In line with this, knockdown of both MIEFs or either of the two genes similarly induces mitochondrial elongation in MEFs (86). However, it was also reported that in HeLa cells, down-regulation of MIEF1 by siRNA resulted in mitochondrial fission (91), while complete knockout of either MIEF alone, or double-knockout of both MIEF1/2 in HeLa cells caused mitochondrial elongation (121). The reasons for these inconsistent results can be that the different cell types used for experiments might have different endogenous levels of the fission/fusion proteins. Furthermore, it has been experimentally shown that introduction of low/intermediate or high levels of MIEF receptor proteins in cells can lead to opposite effects as described in detail below, possibly due to competitive Drp1 binding to the different receptor populations available (122).

**Figure 3:**
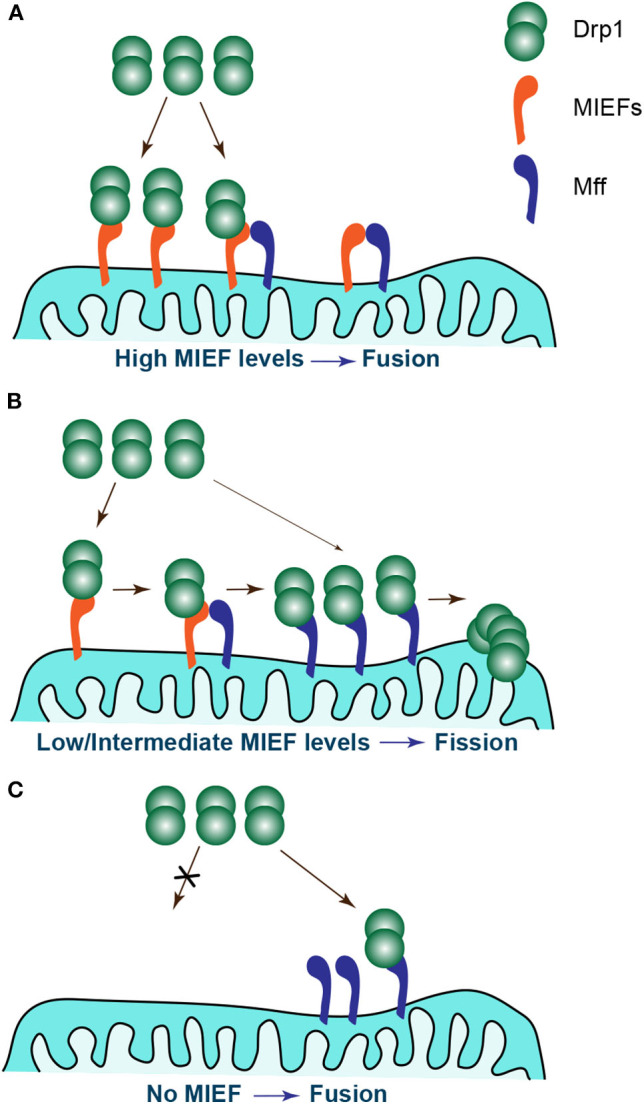
Different levels of MIEFs coordinate with Mff to regulate the balance of mitochondrial dynamics. **(A)** At high levels of MIEFs, Drp1 is sequestered in Drp1-MIEF-Mff and/or Drp1-MIEF complexes on mitochondria, inhibiting a direct Drp1-Mff interaction and resulting in mitochondrial fusion. **(B)** At moderate levels of MIEFs, Mff receives sufficient Drp1 facilitating a direct Drp1-Mff interaction via MIEFs acting as a molecular bridge, and a normal balance of mitochondrial dynamics is maintained. **(C)** In the absence of MIEFs, Mff cannot directly capture sufficient Drp1 from the cytosol by itself, thus the balance shifts to mitochondrial fusion, resulting in mitochondrial elongation.

It should be stressed, however, that the four Drp1 receptors Mff, MIEF1, MIEF2, and hFis1 are usually expressed simultaneously in mammalian cells. Moreover, each receptor is able to independently interact with and recruit Drp1 to mitochondria. This seems to imply a redundant mode of action for the Drp1 receptors in mammals, but these receptors appear to have an additive effect on Drp1 accumulation on mitochondria. However, whether these receptors work coordinately in the process of Drp1 recruitment is largely unclear. A recent study by Yu et al. reports that MIEFs can interact with both Drp1 and Mff and act as adaptors linking Drp1 and Mff in a trimeric protein complex, thereby facilitating a direct interaction between Mff and Drp1 (122). Ablation of both MIEFs largely reduces the association of endogenous Mff with Drp1, and also greatly impairs the Drp1 recruitment and accumulation on mitochondria mediated by Mff overexpression (122). This indicates that endogenous MIEFs regulate Mff-mediated mitochondrial recruitment of Drp1, although Mff also can serve as an independent receptor for Drp1. In contrast, depletion of endogenous Mff does not affect the association of MIEFs with Drp1 and neither has any evident impact on MIEF overexpression-induced recruitment and accumulation of Drp1 on mitochondria, indicating that Drp1 recruitment by MIEFs occurs independently of Mff (122). However, increased levels of MIEFs can decrease the association between Drp1 and Mff, and conversely, elevated expression of Mff reduces the interaction of MIEFs with Drp1. These results suggest that Mff and MIEFs compete for the binding to Drp1. Based on this, we have proposed a working model to illustrate how MIEFs and Mff coordinately balance mitochondrial dynamics (Figure 3). When higher levels of MIEFs exist in cells, Drp1 is sequestered in Drp1-MIEF-Mff and/or Drp1-MIEF complexes, inhibiting a direct Drp1-Mff binding and resulting in mitochondrial fusion (Figure 3A). When MIEFs are present at moderate levels in cells, Mff can receive sufficient Drp1 via MIEFs acting as a molecular bridge facilitating a direct interaction between Mff and Drp1, thereby maintaining normal and balanced mitochondrial morphology (Figure 3B). However, in the absence of MIEFs, Mff cannot directly capture sufficient Drp1 from the cytosol by itself, thus the balance shifts to mitochondrial fusion, resulting in mitochondrial elongation (Figure 3C).

In addition to the interplay between MIEFs and Mff regulating Drp1 recruitment as mentioned above, accumulating evidence suggests that MIEFs play a role in regulating hFis1-induced mitochondrial fragmentation (91, 114). It is reported that MIEFs exhibit a robust interaction with hFis1, in a manner independent of the interaction between MIEFs and Drp1. Thus, elevated levels of MIEFs partially reverse hFis1-induced mitochondrial fragmentation, whereas increased levels of hFis1 reduce the interaction between Drp1 and MIEF1 or MIEF2, respectively, and also partially reverse MIEF overexpression-induced mitochondrial elongation (91, 114). However, it is still largely unclear how MIEFs and hFis1 can regulate mitochondrial morphology via their mutual interactions.

Overall, the recruitment of cytosolic Drp1 to mitochondria is a key step in Drp1-mediated fission. Due to the simultaneous existence of four Drp1 receptors on mitochondria, the regulation of Drp1 recruitment and subsequent fission induction appears to be subject to a much more complex regulation in mammals than in yeast. Therefore, it will be an important topic for future research to further explore the molecular mechanisms mediating the interplay between different Drp1 receptors, in order to better understand their functions in the regulation of mitochondrial dynamics in mammals.

## Crosstalk Between the Mitochondrial Fission and Fusion Machineries

Mitochondrial morphology is dynamically regulated through the two counteracting apparatuses on mitochondria, i.e., the fission and fusion machineries. It is generally believed that the pro-fission proteins (such as Drp1, Fis1, Mff, and MIEFs) and the pro-fusion proteins (such as Mfn1, Mfn2, and OPA1) separately work in the fission and fusion machineries. However, emerging evidence suggests a crosstalk between the fission and fusion machineries. For example, it was reported that Drp1 colocalizes with Mfn2 and Bax at mitochondrial fission sites during apoptosis (123). Interestingly, another study suggests that Drp1 can interact with Mfn2 on the mitochondrial surface and overexpression of Drp1 facilitates mitochondrial tethering and fusion in Mfn2- or Mfn1-deficient cells, indicating that Drp1 participates in mitochondrial fusion in addition to acting as a pro-fission protein (124). Anand et al. provide additional evidence for a crosstalk between the fission and fusion machineries through studying the actions of the long and short forms of OPA1 in mitochondrial dynamics. The short forms (produced by processing) of OPA1 are found to trigger mitochondrial fragmentation and colocalize with the fission machinery, suggesting that the processing of OPA1 can regulate the balance of mitochondrial fission and fusion (61).

**Figure 4:**
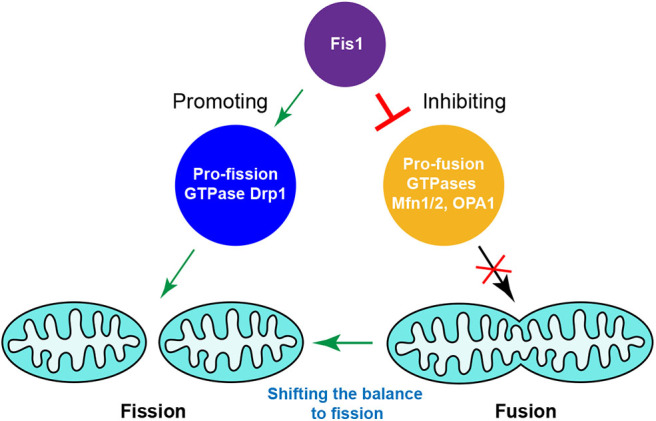
Fis1-mediated crosstalk between the mitochondrial fission and fusion machineries in mammals. Fis1, as a pro-fission factor, is not limited to promoting mitochondrial fission, simultaneously inhibiting the activities of pro-fusion GTPases to shift the balance to fission.

It was recently observed that hFis1 overexpression can induce extensive mitochondrial fragmentation even in the absence of Drp1 or/and Dyn2 (88). This indicates that Drp1/Dyn2 is largely dispensable for hFis1-induced mitochondrial fragmentation, i.e., the existence of Fis1-dependent but Drp1/Dyn2-independent mitochondrial fission pathways in mammals. hFis1 was found to robustly interact with the pro-fusion GTPases Mfn1, Mfn2, and OPA1 at endogenous levels, which supports the idea that hFis1 regulates mitochondrial dynamics via affecting the fusion machinery. In line with this notion, hFis1 reduces the GTPase activity of Mfn1, Mfn2, and OPA1 *in vitro*. Overexpression of hFis1 reduces mitochondrial fusion, whereas knockdown of hFis1 enhances exchange of the matrix content between mitochondria regardless of whether Drp1 is present or absent in cells. Disruption of the fusion machinery by CRISPR-Cas9 genome editing-based knockout technology phenocopied the hFis1 overexpression-induced mitochondrial fragmentation phenotype. Overall, several lines of evidence suggested that hFis1 can induce mitochondrial fragmentation by inhibiting the activity of the mitochondrial fusion machinery (88). This work emphasizes the importance of hFis1 in bidirectional regulation of the mitochondrial fission and fusion machineries and highlights that hFis1, known as a pro-fission factor, is not limited to promoting mitochondrial fission; it can also actively inhibit mitochondrial fusion (Figure 4).

Collectively, all these data provide evidence for a functional crosstalk between the mitochondrial fission and fusion machineries, and suggest that proteins known as components of the fission machinery may act concurrently in the fusion machinery, and likewise, fusion proteins may also affect the fission machinery. While many details remain largely unknown, the bidirectional regulation of mitochondrial morphology through functional interactions between fission and fusion proteins represents an emerging field of research in mammalian mitochondrial dynamics. Supporting this notion, a recent study reveals that the fission and fusion machineries are present at the same ER-mitochondria membrane contact sites to regulate mitochondrial morphology (125).

## Roles of the Endoplasmic Reticulum in Mitochondrial Dynamics

The endoplasmic reticulum (ER) communicates with mitochondria through mitochondria-associated ER membranes (MAMs) in yeast and mammals, and these connections have been shown to participate in different physiological functions, such as phospholipid synthesis, Ca^2+^-mediated signal transduction, protein import, mitochondrial distribution and mitophagy (126–128). It is reported that some of the mitochondria-shaping proteins are involved in this ER-MAM intercommunication. The mitochondrial profusion protein Mfn2 is localized on both mitochondria and the ER, and Mfn2 on the ER associates with Mfn1/2 on mitochondria and tethers the ER and mitochondria together to maintain efficient mitochondrial Ca^2+^ uptake, which is essential for ATP production (47).

The ER also plays an active role in mitochondrial division. ER tubules were observed to wrap around mitochondria, mark the prospective sites of mitochondrial division and reduce the mitochondrial diameter by about 30% before Drp1 recruitment. The ER tubules mainly occur at positions of Drp1 and Mff foci on mitochondria. In fact, ER tubules also mark positions of mitochondrial constriction in the absence of Mff or Drp1 (129). In addition to Drp1 and Mff, two other receptors of Drp1, i.e., MIEF1 and MIEF2, are also observed at mitochondria-ER contact sites, and co-localized with other fission proteins, such as Drp1 and Mff. However, <40% of observed mitochondria-ER contacts at MIEF foci are constriction sites, implicating that MIEFs are not the essential factors to determine ER-mitochondria constriction sites (120). Furthermore, MIEFs require the presence of Drp1 to form foci, whereas Mff can form foci in cells lacking Drp1 (118, 129). Another known Drp1 receptor, Fis1, has been shown in *C. elegans* to enter into a complex containing Drp1, Mff, and ER proteins at the ER-mitochondrial interface during stimulation of mitophagy (112). Strikingly, it is recently reported that endoplasmic reticulum–associated degradation (ERAD), an ER quality control mechanism, regulates mitochondrial dynamics (130). Additionally, in human cells, a subset of ER-mitochondria contacts spatially couple mtDNA replication with downstream mitochondrial division (131). However, it is still unclear to what extent ER tubules are essential for mitochondrial fission and fusion.

## Roles of the Actin Cytoskeleton in Mitochondrial Dynamics

Besides the ER, the actin cytoskeleton is also involved in regulating mitochondrial fission (40, 132). The cycling of actin assembly and disassembly around mitochondrial subpopulations efficiently promotes local Drp1-dependent mitochondrial fission. However, inhibiting Drp1 activity by transfection of Drp1-K38A does not affect actin cycling onto mitochondrial subpopulations as observed through live-cell imaging (133). Furthermore, some actin-related proteins play important roles in mitochondrial fission. An ER-localized actin regulator, INF2 (inverted formin 2) induces actin filaments to drive the initial mitochondrial constriction and then promotes Drp1 recruitment to ER-mitochondria constriction sites (134). An isoform of the actin-nucleating protein Spire1C, localized to the MOM, interacts with INF2, promoting actin assembly at the mitochondrial surface. Disturbing Spire1C or its formin-binding activities can affect mitochondrial constriction and further mitochondrial fission (135). Myosin II plays similar roles in mitochondrial fission, it is enriched at mitochondrial constriction sites, and deletion of Myosin II reduces Drp1 accumulation on mitochondria (136). Interestingly, silencing of the cytoskeletal component septin 2 (Sept2) also decreases the association of Drp1 with mitochondria and increases the average distance between Drp1 clusters (137). In addition, knockdown of the actin-regulating factors cortactin, cofilin, or the Arp2/3 complexes results in elongated mitochondria and disassembles mitochondrial F-actin around mitochondrial subpopulations (133, 138).

The proposed models of mitochondrial fission in mammalian cells are summarized in Figure 2B. Mitochondria are pre-constricted by the ER, together with actin filaments that associate with mitochondria by the action of Spire1C and with the ER by INF2. At the ER constriction site, Drp1 is recruited by its receptors to mitochondria and assembled to form higher-order oligomers around the mitochondrial surface. Then mitochondria are further constricted by Drp1 oligomers, and Dyn2 is recruited to the constricted site instantly to finalize the scission of mitochondria through GTP hydrolysis. In summary, it is a great challenge to understand how the ER, actin cytoskeleton and Drp1 receptors interplay and coordinate their actions to mediate mitochondrial division.

## Roles of Membrane Phospholipids in Mitochondrial Dynamics

In addition to the roles of the ER and actin cytoskeleton in regulating mitochondrial dynamics, mitochondrial membrane phospholipids also communicate with the fusion/fission machinery. Phospholipids are the fundamental building blocks of mitochondrial membrane bilayers. Emerging evidence indicates that at least two types of phospholipids, phosphatidic acid (PA) and cardiolipin (CL), play important roles in mitochondrial dynamics. PA is transported mainly from the ER to the mitochondrial inner membrane, where PA can be converted to CL via multiple enzymatic reactions (139). Some of the CL synthesized in the inner membrane can also be transported to the outer membrane. It has been reported that the pro-fission GTPase Drp1 on the outer membrane directly interacts with CL, and the B insert (also called the variable domain) of Drp1 is responsible for its interaction with CL (140, 141). This interaction between Drp1 and CL stimulates both oligomerization and GTPase activity of Drp1, enhancing mitochondrial division (82, 142, 143). In the outer membrane, CL can be converted back to PA by the MOM-localized phospholipase D (MitoPLD) (144). Drp1 also interacts with PA in the outer membrane, which restrains Drp1 activity, leading to mitochondrial fusion (142). Along these lines, MitoPLD interacts with Drp1, Mfn1, and OPA1, inhibiting Drp1-mediated fission and promoting mitofusin-dependent fusion (142, 144). There is evidence showing that CL located in the inner membrane is directly involved in OPA1-mediated fusion (145), while PA that is generated through the cleavage of CL by MitoPLD in the outer membrane participates in Mfn-mediated fusion (144). Moreover, overexpression of MitoPLD induces mitochondrial fusion, while its knockdown inhibits fusion resulting in mitochondrial fragmentation (144). In line with this, overexpression of lipin 1b [a phosphatase that dephosphorylates PA to form diacylglycerol (DAG)] results in mitochondrial fragmentation (142, 146). Similarly, overexpression of PA-PLA_1_ (PA-preferring phospholipase A1), which hydrolyzes PA to produce lysoPA, triggers mitochondrial fragmentation, whereas knockdown of PA-PLA_1_ induces elongation (147). CerS6-derived sphingolipids interact with Mff and promote mitochondrial fragmentation (148).

Overall, it has become increasingly clear that the interactions between the fusion/fission proteins and the phospholipids in mitochondrial membranes add another layer of complexity to the regulation of mitochondrial fusion-fission dynamics and contributes significantly to our understanding of this process (149–151).

## DRP1-Independent Mitochondrial Fission Pathways in Mammals

**Figure 5:**
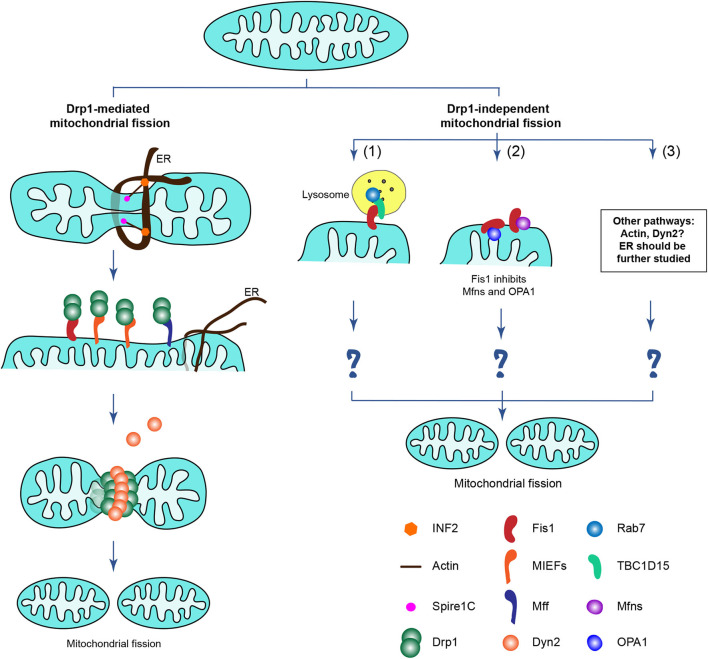
Proposed potential mechanisms driving mammalian mitochondrial fission, including Drp1-dependent and Drp1-independent mitochondrial fission. **(Left)** The Drp1-dependent mitochondrial fission as described in Figure 2B, including Drp1 and its four mitochondrial receptors Fis1, Mff, MIEF1, and MIEF2. **(Right)** Apart from the canonical Drp1-dependent mitochondrial fission, there may be multiple Drp1-independent mechanisms contributing to mitochondrial division in mammals although little is known about their details in the regulation of mitochondrial division. Emerging evidence implies that the Drp1-independent mitochondrial fission may involve: (1) Fis1 together with TBC1D15 and Rab7 regulate both mitochondrial and lysosomal dynamics, which likely is important in mitophagy; (2) Fis1 binds to the pro-fusion GTPases Mfn1/2 and OPA1 and inhibits the activity of the fusion machinery, thereby shifting mitochondrial morphology to a fission phenotype, probably via Drp1-dependent and -independent mechanisms (the details in this process still remain poorly understood); (3) The actin cytoskeleton and Dyn2, as well as the ER may have Drp1-independent functions in regulating mitochondrial division (the details are unclear).

Accumulating data suggest that apart from the canonical Drp1-dependent mitochondrial fission, there may be additional Drp1-independent mechanisms that contribute to mitochondrial division in mammalian cells, as partly described above (Figure 5). Several previous studies show that Fis1 is involved in such a Drp1-independent mitochondrial fission pathway. Onoue et al. reports that Fis1 interacts with TBC1D15 (a GTPase activating protein for the small GTPases Rab7 and Rab11) and recruits the cytosolic TBC1D15 to mitochondria. Fis1, together with TBC1D15, regulates mitochondrial morphology independently of Drp1 (152). This finding is further supported by a recent study showing that Fis1 mediates crosstalk between mitochondria and lysosomes via recruiting TBC1D15 to the mitochondrial surface, and in turn TBC1D15 interacts with lysosomal GTP-bound Rab7 at mitochondria-lysosome contact sites to drive hydrolysis of Rab7-GTP, thereby regulating both mitochondrial and lysosomal dynamics independently of Drp1 (87, 153). Although these findings that the Fis1-TBC1D15-Rab7 interaction involves mitochondria-lysosome contact sites can largely promote our understanding of how Fis1 functions in regulating mitochondrial dynamics, it still seems difficult to fully understand the mechanisms by which Fis1 overexpression induces extensive mitochondrial fragmentation.

In a more recent study mentioned above, hFis1 overexpression was reported to induce extensive mitochondrial fragmentation regardless of the presence or absence of Drp1 (88), further suggesting that Drp1 is dispensable for hFis1-mediated mitochondrial fragmentation. Further analysis showed that hFis1 binds to and inhibits the pro-fusion GTPases Mfn1/2 and OPA1, thereby reducing the activity of the fusion machinery in Drp1-deficient cells, shifting mitochondrial morphology to a fission phenotype (88). Given the importance of the Fis1-TBC1D15-Rab7 involvement in a Drp1-independent mitochondrial fission pathway, ablation of TBC1D15 would be expected to rescue the hFis1 overexpression-induced fragmentation phenotype, but this still remains to be explored. Thus, the underlying mechanisms by which hFis1 can induce mitochondrial fragmentation in the absence of endogenous Drp1 remains to be further explored.

The ER and the actin cytoskeleton have been reported to be involved in mitochondrial dynamics in cooperation with Drp1 (129, 132, 134), but whether the ER and actin cytoskeleton play a role in Drp1-independent fission is unknown. Emerging evidence, however, suggests that F-actin depolymerization significantly prevents hFis1 overexpression-induced mitochondrial fragmentation in a Drp1-independent manner, implying that the actin cytoskeleton plays a Drp1-independent role in regulating mitochondrial fission (88). The ER is generally considered to be the first step of Drp1-mediated mitochondrial fission, promoting mitochondrial constriction before Drp1 recruitment (129), but whether the ER induces mitochondrial scission directly, independently of Drp1 needs to be further explored.

Dyn2 has been reported to work at the final step of Drp1-dependent mitochondrial scission (94), but another study suggests that Drp1-mediated mitochondrial division still occurs in Dyn2-deficient cells (95), indicating Dyn2 is not essential for Drp1-dependent fission. However, a recent report presents that Dyn2 knockdown partially reversed the mitochondrial fission phenotype that was induced when both fission and fusion machineries had simultaneously been destroyed by double knockout of Drp1 and OPA1 through CRISPR/Cas9 technology (88), indicating that Dyn2 may act via a separate mechanism to promote mitochondrial fission when Drp1-mediated fission is blocked.

Collectively, it is becoming increasingly clear that there may be multiple Drp1-independent mechanisms regulating mitochondrial division in mammalian cells. Although substantial progress has been achieved through studies of Drp1-mediated mitochondrial fission, little is known about the regulation of mitochondrial division in mammalian cells lacking Drp1. Undoubtedly, elucidating the underlying mechanisms that drive Drp1-independent mitochondrial division is of fundamental importance to fully understand mammalian mitochondrial dynamics.

## Crosstalk Between Mitochondrial Dynamics and Other Cellular Processes

Accumulating evidence indicates that the carefully orchestrated balance of mitochondrial fusion and fission is critical for maintaining a healthy population of mitochondria. Fission allows the exclusion of damaged mitochondria via mitophagy and mitochondrial biogenesis, whereas fusion allows mitochondria to exchange their substance including proteins and mtDNA among individual mitochondria (154). Abnormal mitochondrial dynamics is directly associated with mitochondrial dysfunction, which impacts on a wide range of cellular processes, such as mitochondrial transport and biogenesis, cell-cycle regulation, cell proliferation and differentiation, energy metabolism, ROS production, Ca^2+^ signaling, mtDNA maintenance, mitochondrial quality control via autophagy (mitophagy), and ultimately programmed cell death (155–160). Moreover, some mitochondrial fission and fusion proteins are known to be critical for embryonic development in mice (45, 161–163), further emphasizing that functions of mitochondrial dynamics go beyond the appearance of mitochondria and have important physiological consequences. In this review, we will focus on the potential roles of mitochondrial dynamics in apoptosis and mitophagy.

### Mitochondrial Dynamics and Apoptosis

Apoptosis, a programmed cell death, occurs in physiological and pathological conditions, and can be activated through intrinsic or extrinsic mechanisms. It is well-established that the Bcl-2 protein family plays crucial roles in the regulation of apoptosis. The Bcl-2 family includes both pro-apoptotic proteins (such as Bax, Bak, Bid, Bad, and Bik) and the anti-apoptotic proteins (such as Bcl-2, Bcl-XL, Bcl-W, and MCL1), which promote and inhibit programmed cell death, respectively (164–167).

Mitochondria have been shown to be involved in the intrinsic apoptotic pathway (168). Emerging data indicate that mitochondrial dynamics participates in the regulation of cell death pathways and remodeling of the mitochondrial network takes place in response to cellular stress, such as hypoxia, drug treatments, and in various pathological conditions. During apoptosis, mitochondria undergo extensive fragmentation and some proteins of the mitochondrial fusion/fission machinery are directly involved in the regulation of apoptosis (169, 170). In general, the prevalent idea is that elongated mitochondria confer cellular resistance to apoptosis, whereas fragmented mitochondria make cells sensitive to apoptotic stimuli (169, 171). For example, knockdown of the pro-fission proteins hFis1 or Drp1 leads to mitochondrial elongation and resistance to different apoptotic stimuli, whereas depletion of the pro-fusion proteins Mfn1, Mfn2, or OPA1 leads to mitochondrial division and makes cells more sensitive to apoptosis (170, 172). Conversely, Mfn2 overexpression reduces sensitivity of cells to radical-induced apoptosis (173). Upon apoptotic stimulation, Drp1 is recruited to the mitochondrial outer membrane, where it co-localizes with Bax and Mfn2 at fission sites, and Drp1 is required for apoptotic mitochondrial fission (104, 123, 174). In agreement with the notion that mitochondrial dynamics regulates the sensitivity of cells to apoptotic stimuli, it was reported that overexpression of MTGM/ROMO1 (a MIM anchored fission protein) results in fragmented mitochondria and triggers a significant release of the death factor Smac/Diablo from mitochondria to the cytosol, but not of other death factors (for instance cytochrome *c*, AIF, or Omi/HtrA2), and does not lead to spontaneous apoptosis. However, down-regulation of MTGM results in elongated mitochondria and increases cell proliferation as well as resistance of cells to apoptotic stimuli (175). Furthermore, it was reported that MIEFs are also involved in the regulation of apoptosis and autophagy. Expression of exogenous MIEF1 induces elevated autophagic activity and decreases the sensitivity of cells to apoptotic stimuli. Conversely, human cells depleted of MIEF1 are more sensitive to apoptotic stimuli (91, 176). However, loss of both MIEF1 and MIEF2 together with Mff in MEFs confers resistance to apoptosis (115). Additionally, Drp1-mediated mitochondrial division controls cristae remodeling through MIEF1/2 during intrinsic apoptosis (121). Moreover, the OMM-associated E3 ubiquitin ligase MARCH5 coupled with MIEF2 controls Drp1-dependent mitochondrial fission and cell sensitivity to stress-induced apoptosis (177).

In healthy cells, Bax is normally localized predominantly in the cytosol, but during apoptosis, Bax translocates from the cytosol to the surface of mitochondria (178), where Bax, in coordination with Bak, results in mitochondrial outer membrane permeabilization (MOMP) (179). Several lines of evidence show that some mitochondria-shaping proteins participate in the Bax translocation. For example, hFis1 is required for translocation of Bax from the cytosol to the surface of mitochondria (104), Drp1 is required for activation and oligomerization of Bax (180, 181), and OPA1 regulates cristae remodeling and triggers the release of cytochrome *c* to the cytosol (182, 183). More recently, MIEF1 was reported to regulate the mitochondrial translocation and oligomerization of Bax via BCL2L1 (also a member of Bcl-2 family), but independently of Drp1 (176).

On the other hand, extensive data suggest that the Bcl-2 family also modulates mitochondrial fusion/fission dynamics (184, 185). Bax/Bak are not just involved in Drp1-mediated mitochondrial fission during apoptosis (186–188), but also regulate normal mitochondrial fusion by affecting Mfn2 in healthy cells (189). Intriguingly, it is reported that Bak can induce mitochondrial fragmentation during apoptosis by differentially interacting with Mfn1 and Mfn2 (190). Bcl-XL interacts with Drp1 to promote Drp1-mediated fission and increases the rates between fission and fusion in normal neurons (191, 192). MCL1 is known to have two splice isoforms, and the long isoform (MCL1L) is anchored to the MOM as an anti-apoptotic protein binding to Drp1, while the short isoform (MCL1S) acts as a pro-apoptotic factor. A lower MCL1L/S ratio inhibits Drp1 translocation from the cytosol to mitochondria, leading to mitochondrial elongation (193).

In addition, mitochondrial membrane phospholipids have been suggested to play a role in the regulation of apoptosis (194), and accumulating evidence suggests that CL acts as an interaction platform to recruit apoptotic factors such as tBid and Bax (195–198). CL is also essential for promoting the association between BAX dimers, hence CL plays an important role in controlling the formation of active BAX oligomers (199). Ceramide and sphingolipid are also reported to be critical for the formation of Bax oligomers during apoptosis (200, 201). A recent study further shows that ceramides interact directly with the voltage-dependent anion channel VDAC2, a mitochondrial platform for Bax/Bak translocation, and trigger mitochondrial apoptosis (202).

Furthermore, emerging evidence implicates that mitochondrial dynamics influences appearance, distribution and stability of mtDNA nucleoids in mitochondrial networks (203). For instance, loss of Drp1 function results in severe mtDNA nucleoid clustering and loss of mtDNA (204–206), and disruption of mitochondrial fusion leads to mtDNA instability (207–209). Interestingly, a recent study reported that increased mitochondrial fission by Drp1 overexpression triggers the release of mtDNA into the cytosol, resulting in cytosolic mtDNA stress in hepatocellular carcinoma (HCC) cells (210). There is also evidence that Bak/Bax macropores facilitate mtDNA release from mitochondria during apoptosis regardless of mitochondrial fusion/fission dynamics (211). Obviously, more work is needed to understand the role of mitochondrial dynamics in these processes.

In summary, mitochondrial fragmentation occurs in most forms of apoptosis through activation of the mitochondrial fission machinery and/or inhibition of the fusion machinery in various physiological and pathophysiological conditions. However, how this process is regulated and the precise mechanisms by which the mitochondria-shaping proteins take part in apoptotic progression is largely unclear. Furthermore, whether Drp1-mediated fission is necessary during apoptosis is still controversial. There are reports suggesting that Drp1-mediated fission is an early step during apoptosis, since Drp1 depletion delays the cytochrome *c* release and subsequent apoptosis (212–214). Conversely, it is suggested that mitochondrial fission and apoptosis are two separate processes, because mitochondrial hyper-fusion caused by down-regulation of Drp1/Fis1 does not prevent cell death after apoptotic stimuli (215, 216). Moreover, it is reported that the mitochondrial network in *DRP1*^−/−^ MEFs undergoes extensive fragmentation during apoptosis (162). Consistent with these data, apoptotic mitochondrial fragmentation still occurs during Bax/Bak-dependent apoptosis in Drp1-deficient cells albeit loss of Drp1 results in mitochondrial network super-fusion (211). Mechanistically, these data suggest that Drp1-independent fission might be involved in mitochondrial fragmentation during apoptosis in addition to canonical Drp1-dependent fission.

### Mitochondrial Dynamics and Mitophagy

The mitochondrial quality control is executed through mitophagy, which selectively removes senescent or damaged mitochondria and balances the overall mitochondrial mass between biogenesis and degradation. Mitophagy is regulated mainly by Parkin and PINK1 in mammals, and mutations in the genes encoding these proteins are associated with Parkinson's disease (217, 218). While Parkin normally exists in the cytosol, when cells lose the mitochondrial membrane potential, such as by carbonyl cyanide m-chlorophenylhydrazone (CCCP) treatment, PINK1 accumulates at the surface of impaired mitochondria and recruits Parkin from the cytosol to mitochondria. Parkin, recruited by PINK1 to the surface of damaged mitochondria, ubiquitylates certain mitochondrial proteins (e.g., Mfn1/2) and promotes engulfment of damaged mitochondria by lysosomes (218, 219). There are a number of Parkin ubiquitylation substrates on the MOM and in the cytosol (220, 221). The mitochondrial outer membrane protein Miro1 (Mitochondrial Rho GTPase 1), essential for mitochondrial transport (222), interacts with Parkin and functions as a Ca^2+^-sensitive docking site for Parkin during mitochondrial damage. Knockdown of Miro1 reduces Parkin translocation to mitochondria and suppresses mitophagy (223).

In addition to the canonical Parkin-dependent mitophagy pathway mentioned above, increasing evidence shows that mitophagy can occur through Parkin-independent pathways, such as other ubiquitin ligase- and receptor-mediated mitophagy processes as well as mitochondrial lipid-mediated mitophagy (224). In addition to Parkin, several other E3 ligases have been shown to be involved in mitophagy, e.g., MUL1, ARIH1, and MARCH5 (225–227). Several mitophagy-specific receptors, such as FUDNC1, NIX, BNIP3, PHB2, and BCL2L13, have been reported to recognize damaged mitochondria, interact with LC3 and recruit autophagosomes decorated by LC3 (228–230). NLRX1 has recently been identified as a new mitophagy receptor in *L. monocytogenes* (231).

Additionally, mitochondrial membrane lipids also play important roles in regulating mitophagy. For instance, ceramide (a bioactive sphingolipid) in the MOM is found to interact with LC3B-II and trigger lethal mitophagy through the selective targeting of LC3B-II-containing autophagosomes to mitochondria (232). It has also been shown that CL externalization (i.e., CL is translocated from the MIM to the MOM) plays an important role in modulating mitophagy. During mitophagic stimulation, the externalized CL can form an essential platform on the surface of mitochondria where CL binds to LC3, and initiates autophagosome formation, ultimately resulting in mitophagy (233). The role of CL in mitophagy is supported by the findings that Beclin 1, a central regulator of mitophagy, directly interacts with CL on the MOM, regulating mitophagy (234). Another study suggests that LC3B, a structural protein of autophagosomal membranes, is translocated to the MOM by interaction with externalized CL (235).

Hypoxia-induced/FUNDC1-mediated mitophagy and mitochondrial dynamics are likewise important. It has been reported that the MOM-anchored protein FUNDC1 drives hypoxia-induced mitophagy through interacting with the key autophagy factor LC3 in mammals (236). Upon mitophagy induced by hypoxia or treatment with FCCP, FUNDC1 binds and recruits ULK1 (a Ser/Thr kinase required for early autophagosome formation) to damaged mitochondria, where FUNDC1 is phosphorylated by ULK1, inducing mitophagy (237). FUNDC1 also interacts with Drp1 and OPA1, and increased association of FUNDC1 with Drp1 and less interaction with OPA1 promote mitochondrial fission and mitophagy (238). FUNDC1 is also found to accumulate at the mitochondria-associated ER membranes (MAMs) and interact with the ER membrane protein calnexin. Under hypoxic conditions, FUNDC1 associated more with Drp1 accompanied with less interaction with calnexin, triggering mitochondrial fission and mitophagy (239). Additionally, Bcl-2 family protein Bcl-xL also participates in FUNDC1-mediated mitophagy. Bcl-xL but not Bcl-2, can inhibit the dephosphorylation of FUNDC1 to block further mitophagy (240).

Growing evidence links mitochondrial dynamics with mitochondrial quality control via selective removal of damaged mitochondria (i.e., mitophagy). The mitochondrial fusion factors OPA1, Mfn1, and Mfn2 are involved in the regulation of Parkin/PINK1-mediated mitophagy. OPA1 mutations or impaired cleavage of OPA1 affect mitophagy and Mfn1 and Mfn2 are ubiquitinated in a PINK1/Parkin-dependent manner upon mitophagic stimulation (241–243). Mfn2, but not Mfn1, was reported to mediate Parkin recruitment to damaged mitochondria. PINK1 can phosphorylate Mfn2, thereby promoting the interaction of Mfn2 with Parkin, the translocation of Parkin to mitochondria and Parkin-mediated ubiquitylation of mitochondrial proteins. Loss of Mfn2 prevents the translocation of Parkin to mitochondria (49). Emerging data suggest that Drp1 and its four receptors also participate in mitophagy. For example, overexpressing exogenous hFis1 in MEF cells triggers mitophagy (244), whereas cells lacking one or more Drp1 receptors (Fis1, Mff, and MIEFs) are resistant to CCCP-induced mitochondrial fragmentation to different degrees (86, 115). However, the resistance to apoptosis resulting from Fis1/Mff depletion can be reversed through overexpression of MIEF1 or MIEF2 (86). Of note, knockdown of MIEF1 by RNAi triggers and overexpression of MIEF1 blocks Parkin recruitment to mitochondria at the early stages of CCCP treatment (176). Interestingly, transient transfection of exogenous MIEF1 without CCCP treatment still leads to elevated expression of LC3B-II, implicating increased mitophagic activity (91). Additionally, some apoptotic factors in the Bcl-2 family are reported to be involved in the Parkin/PINK1-mediated mitophagic process. Bcl-xL, MCL1, and Bcl-W, but not Bcl-2, Bax, and Bak can inhibit this type of mitophagy (167).

Additionally, the SNARE protein syntaxin 17 (STX17), which is involved in the process of autophagy, is shown to cooperate with mitochondria-shaping proteins in mitophagy (245, 246). Intriguingly, it is reported that loss of Fis1 leads to aberrant accumulation of STX17 on mitochondria and induces mitophagy in Fis1-deficient cells in a way independent of Parkin/PINK1 (247). Furthermore, STX17 also localizes at the ER-mitochondria contact sites and regulates the localization and GTPase activity of Drp1 (248, 249). These data support the notion of an intricate crosstalk between autophagy factors and mitochondria-shaping proteins during mitophagy. Overall, although a number of mitochondria-shaping proteins are implicated in mitophagy, the detailed roles of these proteins in the process remain poorly understood. A deeper understanding of these mechanisms will be of major importance especially in the field of neurodegeneration.

## In Summary

In the past 20 years, considerable progress has been made in terms of identifying core components and regulatory factors of the mitochondrial fission/fusion machineries in mammals, and huge efforts have been made to elucidate the potential molecular mechanisms that control and regulate mitochondrial fission and fusion events. One outcome of these efforts is a shift from the view that the mitochondrial fission and fusion machineries are evolutionarily conserved from yeast to human, to an understanding that these processes are much more complicated in mammals than in single-cell yeast. So far, at least four Drp1 receptors (Fis1, Mff, MIEF1, and MIEF2) in mammalian cells are responsible for recruitment of Drp1 to mitochondria with an additive effect, although they are not functionally redundant. Increasing evidence indicates that these receptors also coordinately work in Drp1-mediated mitochondrial fission. Thus, further research is warranted to elucidate the interplay between these Drp1 receptors in health and disease. Although the pro-fission and pro-fusion proteins are thought to regulate mitochondrial fission and fusion events separately, emerging data suggest that there is also extensive crosstalk between the fission and fusion machineries. It is also becoming increasingly clear that there are additional Drp1-independent mechanisms that drive mitochondrial division in mammalian cells. Finally, research into the mechanisms of apoptosis and mitophagy has excitingly placed the mitochondrial fission and fusion machineries at the center of these physiological processes. Elucidation of the underlying molecular mechanisms involved in these processes will be critical for better understanding of mammalian mitochondrial dynamics.

## Author Contributions

RY and JZ conceptualized and wrote the review with contributions from MN and UL. RY and JZ made the figures. All authors discussed and approved the final manuscript.

## Conflict of Interest

The authors declare that the research was conducted in the absence of any commercial or financial relationships that could be construed as a potential conflict of interest.
